# The effect of transcranial alternating current stimulation on functional recovery in patients with stroke: a narrative review

**DOI:** 10.3389/fneur.2023.1327383

**Published:** 2024-01-10

**Authors:** Seoyon Yang, You Gyoung Yi, Min Cheol Chang

**Affiliations:** ^1^Department of Rehabilitation Medicine, School of Medicine, Ewha Woman's University Seoul Hospital, Seoul, Republic of Korea; ^2^Department of Physical Medicine and Rehabilitation, College of Medicine, Yeungnam University, Taegu, Republic of Korea

**Keywords:** transcranial alternating current stimulation, stroke, treatment, rehabilitation, review

## Abstract

Stroke is a common neurological disorder worldwide that can cause significant disabilities. Transcranial alternating current stimulation (tACS) is an emerging non-invasive neuromodulation technique that regulates brain oscillations and reshapes brain rhythms. This study aimed to investigate the effect of tACS on functional recovery in patients with stroke. The MEDLINE (PubMed), Cochrane Library, Embase, SCOPUS, and Web of Science databases were searched for English-language articles on tACS and stroke, published up to October 20, 2023. The following key search phrases were combined to identify potentially relevant articles: ‘tACS,’ ‘transcranial alternating current stimulation,’ ‘stroke,’ ‘cerebral infarct,’ and ‘intracerebral hemorrhage.’ The inclusion criteria for study selection were as follows: (1) studies involving patients with stroke and (2) studies that used tACS for functional recovery. A total of 34 potentially relevant studies were identified. Five articles were included in this review after reading the titles and abstracts and assessing their eligibility based on the full-text articles. Among the included studies, one investigated the improvement in overall functional status in patients with stroke after tACS, and two investigated the effect of tACS on motor function and gait patterns. Moreover, one study reported the efficacy of tACS on aphasia recovery, and one study evaluated the effect of tACS on hemispatial neglect. Our findings suggest that tACS improves functional recovery in patients with stroke. The application of tACS was associated with improved overall functional recovery, sensorimotor impairment, aphasia, and hemispatial neglect. The potential clinical application of tACS should be supported by high-quality, evidence-based studies.

## Introduction

Stroke is a common neurological disorder that occurs worldwide and causes significant disability (1). Patients with stroke have neurological deficits in different functional domains that can be permanent (2). Following a stroke, patients can have various sequelae, such as motor impairments, sensory loss, visual field defects, cognitive impairments, dysphagia, and language impairment (3). Such functional problems that persist after stroke can have a substantial negative impact on the quality of life and result in emotional stress. Rehabilitation therapy is crucial for ameliorating severe dysfunction resulting from strokes; it assists patients in regaining full functionality and reintegrating into their daily routines (4).

Normally, functional balance between the two hemispheres of the brain is achieved through interhemispheric inhibition (5). In patients with stroke, brain damage caused by the events leads to abnormal increases in interhemispheric inhibition and enhanced excitability of the contralesional hemisphere (6). Cortico-subcortical excitability and neural network changes can cause severe functional disabilities (7). Among the various rehabilitation methods, non-invasive brain stimulation (NIBS) modulates cortical excitability and helps regain balance between the two hemispheres (8). Moreover, NIBS aims to induce neuroplasticity and facilitate recovery by modulating neural processing. Repetitive transcranial magnetic stimulation (rTMS), transcranial direct-current stimulation (tDCS), and transcranial alternating current stimulation (tACS) are commonly used NIBS methods to induce a better recovery (9). rTMS activates axons through short-pulsed stimulation by inducing new action potentials, whereas tDCS manipulates the membrane potential of neurons and modulates spontaneous firing rates (10).

tACS is an emerging NIBS method used to regulate brain oscillations and reshape brain rhythms (11). Sinusoidal alternating electric currents are delivered to the head via scalp electrodes in specific brain regions to modulate brain activity. The duration of stimulation, frequency, amplitude, phase difference, and the site of stimulation are major parameters used in the application of tACS (12). The intensity of the alternating-current is provided within the range of 0.5–2 mA using metal or rubber electrodes with a skin-electrolyte contact area of 25–35 cm^2^ (13). The electrical current alternates between two electrodes (a positive electrode is called an anode, and a negative electrode is called a cathode) back and forth as a sinusoidal wave. These weak and constant direct electrical currents affect cortical neurons and alter cortical excitability (14). Furthermore, tACS is believed to improve brain function by modulating the intrinsic oscillatory activity in a frequency-dependent manner (15).

Previous studies have investigated the relationship between tACS-induced neural oscillations and improvements in behavioral and cognitive functions (16). The networks of oscillatory activity are classified into frequency bands (delta-δ: 1–3 Hz; theta-θ: 4–7 Hz; alpha-α: 8–13 Hz; beta-β: 14–30 Hz; gamma-γ: 30–80 Hz; fast, 80–200 Hz; ultra-fast, 200–600 Hz) (17). The associations of alpha-band oscillations (8–13 Hz) with visual task performance (18), beta-band oscillations (14–30 Hz) with motor performance (19), and theta-band oscillations (4–7 Hz) with memory (20) have been reported. Therefore, tACS could be an effective therapeutic tool to enhance stroke rehabilitation. However, no previous studies have summarized the effect of tACS on functional recovery in patients with stroke. This review investigated tACS therapy for functional recovery in patients with stroke.

## Methods

The MEDLINE (PubMed), Cochrane Library, Embase, SCOPUS, and Web of Science databases were searched for English-language articles about tACS and stroke, published up to October 20, 2023. The following key search phrases were combined to identify potentially relevant articles: ‘tACS,’ ‘transcranial alternating-current stimulation,’ ‘stroke,’ ‘cerebral infarct,’ and ‘intracerebral hemorrhage’ (Table 1). The inclusion criteria for study selection were as follows: (1) studies involving patients with stroke and (2) studies which used tACS for functional recovery. The exclusion criteria were as follows: (1) reviews, (2) case reports, (3) commentaries, (4) letters, (5) animal studies, and (6) study outcomes that were either insufficient or not reported.

**Table 1:** Search terms and strategies.

Database	Keywords
MEDLINE	(“stroke”[All Fields] OR “cerebral infarct”[All Fields] OR “intracerebral hemorrhage”[All Fields]) AND (“tACS”[All Fields] OR “transcranial alternating current stimulation”[All Fields])
Cochrane library	#1 “stroke” OR “cerebral infarct” OR “intracerebral hemorrhage” 84,173 #2 “tACS” OR “transcranial alternating current stimulation” 3,397 #3 #1 and #2 in Trials 112
Embase	(‘stroke’/exp. OR ‘stroke’ OR ‘cerebral infarct’/exp. OR ‘cerebral infarct’ OR ‘intracerebral hemorrhage’/exp. OR ‘intracerebral hemorrhage’) AND (‘tacs’ OR ‘transcranial alternating current stimulation’/exp. OR ‘transcranial alternating current stimulation’)
Scopus	ALL ((“stroke” OR “cerebral infarct” OR “intracerebral hemorrhage”) AND (“tACS” OR “transcranial alternating current stimulation”))
Web of Science	ALL = ((“stroke” OR “cerebral infarct” OR “intracerebral hemorrhage”) AND (“tACS” OR “transcranial alternating current stimulation”))

## Results

**Figure 1:**
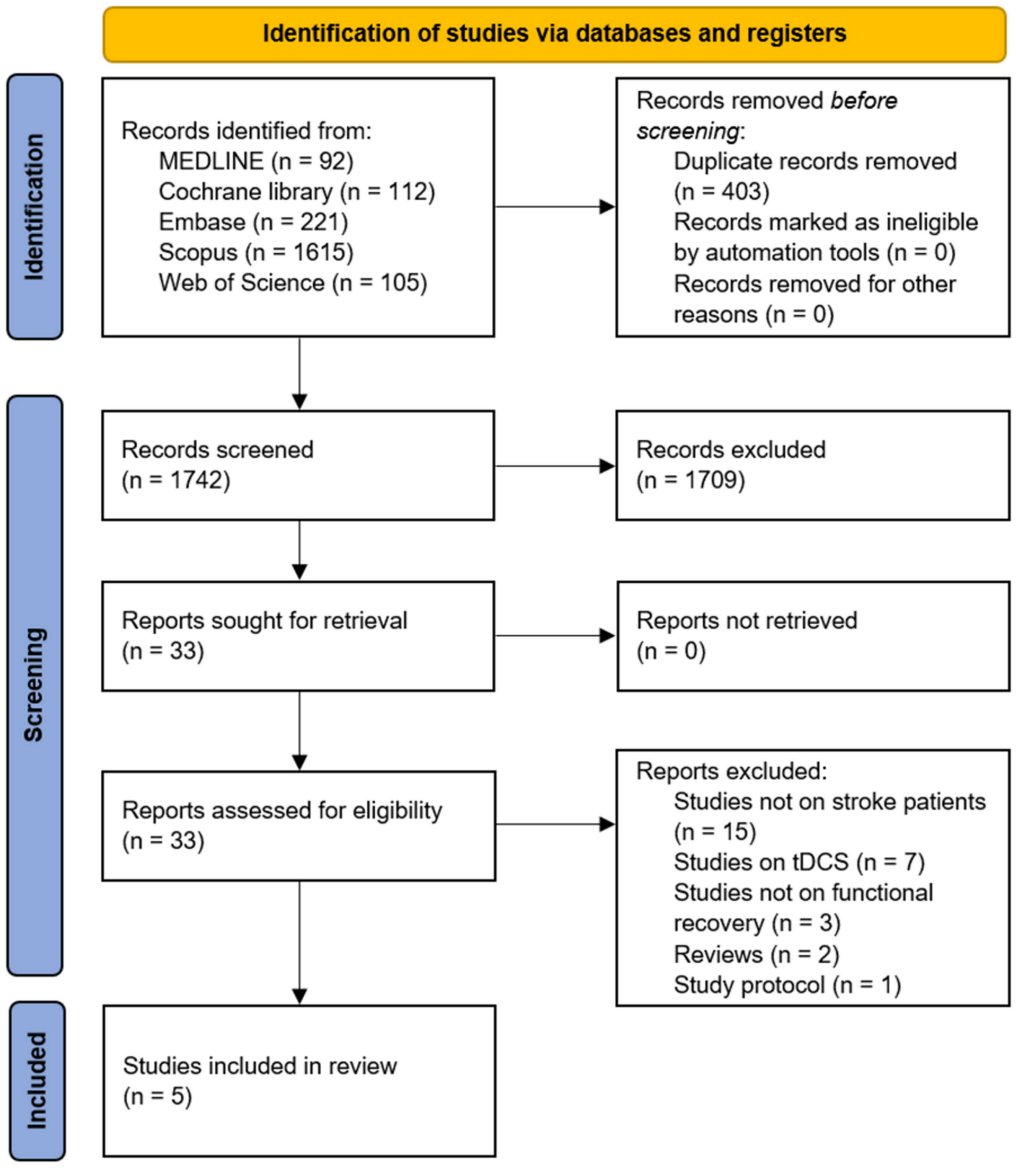
Flow diagram of the study selection process.

After the search, 1,742 potentially relevant articles were identified. The titles and abstracts of the articles were screened, and their eligibility was assessed based on the full-text articles. Five studies were included in this review (Figure 1). Details of the included articles are presented in Table 2. Among the included studies, only one study highlighted an improvement in overall functional status in patients with stroke after tACS (21), and two studies investigated the effect of tACS on motor function and gait patterns (22, 23). One study reported the efficacy of tACS on aphasia recovery (24), whereas one study evaluated the effect of tACS on hemispatial neglect (25).

**Table 2:** Characteristics of included studies.

#	First author	Year	Study design	No. of patients (active/ control)	Patients	Site	Intensity (mA)	Duration (min)	No. of sessions	Outcome parameters	Results
1	Wu et al. (21)	2016	RCT	60 (30 tACS vs. 30 usual rehabilitation)	Subacute stroke between 15 and 60 days after the onset	Bilateral mastoids	20 Hz	30 min	15 sessions	Overall functional status (NIHSS score, mean blood flow velocity (MFVs), Gosling pulsatility index (PI))	The mean NIHSS scores and Gosling PI of the the tACS group demonstrated a significant decrease compared to the control group.
2	Kitatani et al. (17)	2020	PCO	8 (tACS and sham)	Single stroke ≥6 m	M1 (foot area)	40 Hz	10 min	10 sessions (tACS-sham crossver)	Gait (EMG)	Gait intervention with tACS was effective in modulating the cortical control of muscle activity during gait and enhancing gait function.
3	Xie et al. (19)	2022	RCT	30 (14 active tACS vs. 11 sham tACS)	Post-stroke aphasia ≥6 m	SMA	6 Hz	30 min	14 sessions	Aphasia (Aphasia Battery of Chinese (ABC))	The results demonstrated that the active tACS plus SLT group exhibited significantly greater improvements in AQ and auditory verbal comprehension than the sham tACS plus SLP group.
4	Yuan et al. (18)	2022	POS	13 (crossover, 10 Hz, 20 Hz, sham)	Unilateral stroke ≥6 m	Ipsilesional M1 (C3/C4) and contralesional supraorbital ridge (FP1/FP2)	10 Hz, 20 Hz	20 min	3 sessions	Motor (Fugl-Meyer Assessment, Action Research Arm Test, functional MRI)	10 Hz tACS mainly modulated FC within motor-related regions and 20 Hz tACS modulated regions beyond the motor-related areas.
5	Schuhmann et al. (20)	2022	POS	16 (crossover, 10 Hz, sham)	Stroke patients with hemispatial neglect	Contralateral posterior parietal cortex	10 Hz	30 min	2 sessions	Hemispatial neglect (CVDT)	Patients who received tACS demonstrated significant improvement in reducing neglect symptoms measured with a CVDT and BT.

BT, Bell’s task; CVDT, computerized visual detection test; EMG, electromyography; LBT, line bisection task; FC, functional connectivity; SMA, supplementary motor area; M1, primary motor cortex; NIHSS, National Institute of Health Stroke Scale.

A randomized controlled trial (RCT) conducted by Wu et al. in 2016 recruited 60 patients with stroke and demonstrated that tACS positively enhanced neurological function (26). Thirty patients received 15 sessions of tACS over the mastoids bilaterally (20 Hz and < 400 μA for 30 min). Mastoid regions, which are close to the subcortical structures around the medulla and cerebellum, are expected to increase cerebral blood flow and induce functional recovery as they are close to the cerebello-hypothalamic projections. The National Institutes of Health Stroke Scale (NIHSS) scores, which reflect the overall functional status of patients with stroke and cerebral hemodynamics using transcranial Doppler, of these patients were compared with those of 30 patients in the control group, who received the usual rehabilitation program only. When patients in the tACS group received the tACS in a quiet treatment room, patients in the control group were asked to sit on a chair for the same period in the same room with tACS. All patients received a standardized rehabilitation program, which included aerobic exercise, daily function training, and speech and cognitive training for 3 h. Patients were unaware of which group they belonged to or what treatment the other group received. The NIHSS is often used to measure neurological function in post-stroke patients and consists of 15 items, including levels of consciousness, visual fields, facial muscle function, extraocular movements, language, speech, motor strength, sensory function, coordination, and hemi-inattention (27). The results demonstrated that the tACS group had a significant decrease in mean NIHSS scores, a larger increase in blood flow velocity, and a decrease in the resistance of the vascular bed compared to the control group. The mechanism of tACS action in stroke recovery is that electrical stimulation to brain tissues enhances cerebral hemodynamics, including global and regional cerebral blood flow (28). This study also showed that tACS increased the cerebral blood flow velocity, which induced recovery of neurological function. Thus, this study concludes that tACS effectively improves sensorimotor function and cerebral hemodynamics in patients with stroke.

In 2020, Kitatani et al. conducted a small single-blind crossover study to investigate the effects of gait-synchronized oscillatory brain stimulation with tACS. Eight patients with chronic stroke received tACS over the ipsilesional primary motor cortex (M1) foot area (40 Hz for 10 sessions, twice a week for 5 weeks). The primary motor cortex is a brain region located anterior to the central sulcus, which is traditionally implicated in voluntary movement control (29). For stimulation over the foot area of M1, the positions of the electrodes were determined to be where the transcranial magnetic stimulation elicited the best motor response in the tibialis anterior muscle. Patients performed a 10-min treadmill walking at a comfortable pace along with tACS, which was synchronized with the individual gait cycle frequency. Sham stimulation was performed in a crossover manner during the gait cycle. The results demonstrated that tACS in gait intervention, particularly targeting β-band (15–35 Hz) coherence, induced gait-specific plasticity and changes in gait function by enhancing the excitability of the cortical control of the paretic tibialis anterior muscle activity during gait.

The effect of tACS on recovery from aphasia in patients with stroke was reported in 2022 (24). Considering that neuronal oscillations in language-related brain areas may also be associated with speech and language processing (30), Xie et al. investigated whether tACS is effective in recovering post-stroke aphasia (24). Twenty-five patients with stroke suffering from aphasia were randomized into the active tACS (n = 14) or sham tACS (n = 11) groups. In the active tACS group, 6 Hz tACS was applied during 30 min of speech-language therapy (SLT) over the supplementary motor area (SMA) for 14 consecutive days. All patients believed that they received active tACS. tACS was delivered for the entire 30-min intervention period and 30-s ramp-up and-down phases in the study group. Patients in the sham group received tACS only during the ramp-up and-down phases. The stimulation site was SMA, which is involved in both speech production and comprehension (31, 32), and tACS over the SMA was expected to enhance the efficacy of SLT. Both groups received 30 min of SLT with active or sham tACS, followed by 90 min of SLT alone (14 sessions). The results demonstrated that in the active tACS plus SLT group the aphasia quotient and auditory verbal comprehension improved significantly more than the sham tACS and SLT group. This study suggests tACS over the SMA as an additive rehabilitative tool to strengthen the effect of SLT in patients with aphasia and stroke.

In 2022, Yuan et al. investigated whether tACS produced differential modulation effects according to frequency. Thirteen patients with chronic stroke were enrolled, and the effects of tACS at different frequencies (10 Hz, 20 Hz, sham) were evaluated using functional magnetic resonance imaging (fMRI). Additionally, tACS was applied over the ipsilesional M1, and 1 mA current was delivered for 20 min at 10 Hz and 20 Hz. At 10 Hz, tACS had a limited effect on motor-related regions, whereas tACS at 20 Hz modulated brain regions beyond motor-related areas. Therefore, this study concludes that applying 20 Hz tACS promotes a heightened functional interplay between the brain regions associated with executive control and the sensorimotor area, surpassing the effects observed with both 10 Hz tACS and sham stimulation.

Moreover, tACS was also effective in improving spatial attention deficits after stroke. In 2022, Schuhmann et al. conducted a crossover study to evaluate whether tACS alleviated attention deficits. Sixteen subacute patients with stroke with visuospatial neglect symptoms were enrolled and received 10 Hz tACS or sham stimulation. Alpha-band oscillations (8–12 Hz) over the posterior parietal cortex is known to be associated with attentional bias. Therefore, tACS at 10 Hz targeting the contralesional posterior parietal cortex was administered for 30 min. Patients who received tACS demonstrated a significant reduction in neglect symptoms, which were measured with a computerized visual detection task and Bell’s task, compared to patients with sham stimulation. Therefore, the potential clinical utility of tACS was proposed for improving hemispatial neglect symptoms.

Four studies did not mention whether there were any side effects of tACS (23–26). Only the study by Kitatani et al. (22) reported that no patients experienced side effects, such as vertigo, skin pain or irritation, headaches, or phosphenes from the stimulation, which are commonly reported adverse effects of tACS (33).

## Discussion

This review explored whether tACS can be considered an alternative treatment option for improving functional disabilities in patients with stroke. Studies included in this review demonstrated that applying tACS produced improvement in overall functional recovery, sensorimotor impairment, aphasia, and hemispatial neglect.

However, the mechanism of action of tACS is not fully understood. We hypothesized that the effects of tACS could be induced by entrainment and spike-timing-dependent plasticity (STDP) (16). Entrainment refers to the synchronization of the stimulated frequency with endogenous neural oscillations. As the stimulated frequency approaches the endogenous frequency of the targeted neural network, it can effectively modulate rhythmic oscillations (34). Additionally, STDP refers to synaptic plastic changes after stimulation that depend on synaptic events’ timing. Synaptic strength increases when presynaptic spikes occur before postsynaptic spikes, whereas the strength is weakened when postsynaptic spikes occur before presynaptic spikes (16). Synaptic strengthening has been assumed to occur if the stimulation frequency is close to the endogenous frequency. In contrast, if the stimulation frequency is higher than the endogenous frequency, a postsynaptic spike occurs before the presynaptic spikes and the synapse may be weakened (16, 35). In addition, as tACS generates a weak oscillating extracellular field in the brain, subthreshold membrane potential shifts can increase or decrease membrane potentials (36). Changes in membrane potential affect spike timing at the single-neuron level and cause depolarization or hyperpolarization, which may trigger neuronal plasticity (37). Thus, tACS may facilitate neuroplasticity, which is essential in recovery (38).

The application of tACS may be recommended, considering the efficacy of stimulation in stroke recovery and its several advantages. First, tACS is usually well-tolerated and is both feasible and inexpensive (25). Moreover, tACS can also be easily applied in the clinical setting. Second, tACS is portable and can be used in a home setting (25). Third, tACS is safe and does not cause any serious adverse events (39). Therefore, tACS appears to be a readily available and safe alternative therapeutic approach to improve brain function. As tACS modulates oscillations and oscillatory connectivity in the brain, it may also be a potentially effective therapeutic tool to enhance brain plasticity, improving overall functional status, sensorimotor deficits, language problems, and attentional deficits.

The studies included in this review had a few limitations. First, the sample sizes of the included studies were small. Studies with large sample sizes are required to investigate the effects of tACS in patients with different functional impairments. Second, only a few studies have investigated the effect of tACS on functional recovery after stroke, and the functional status of patients with stroke differed across studies. Further studies on the effects of tACS on stroke recovery are warranted. Third, the long-term effects of tACS were not reported in these studies. Further studies investigating the clinical efficacy of tACS, including repeated treatment sessions and evaluating the long-term effects of stimulation, are recommended.

## Conclusion

Our findings suggest that tACS improves functional recovery in patients with stroke. The application of tACS was associated with improved overall functional recovery, sensorimotor impairment, aphasia, and hemispatial neglect. The potential clinical application of tACS should be supported by high-quality, evidence-based studies.

## Author contributions

SY: Conceptualization, Data curation, Formal analysis, Investigation, Methodology, Software, Validation, Visualization, Writing – original draft, Writing – review & editing. YY: Conceptualization, Data curation, Formal analysis, Investigation, Methodology, Software, Validation, Visualization, Writing – original draft, Writing – review & editing. MC: Conceptualization, Data curation, Formal analysis, Investigation, Methodology, Software, Supervision, Validation, Visualization, Writing – original draft, Writing – review & editing.
